# Risk factors associated with severe manifestations of 2009 pandemic influenza A (H1N1) infection in China: a case–control study

**DOI:** 10.1186/1743-422X-10-149

**Published:** 2013-05-15

**Authors:** Yan-yan Ren, Yu-yan Yin, Wen-qing Li, Yi Lin, Ti Liu, Shuang Wang, Sheng-yang Zhang, Zhong Li, Xian-jun Wang, Zhen-qiang Bi

**Affiliations:** 1 Shandong Center for Disease Prevention and Control, Jinan, Shandong Province, China; 2 Shandong Provincial Key Laboratory of Infectious Disease Control and Prevention, Jinan, Shandong Province, China; 3 Shandong Institute for Endemic Diseases Control and Research, Jinan, Shandong Province, China; 4 Brigham and Women’s Hospital, Harvard Medical School, Boston, MA, USA; 5 Shandong University School of Public Health, Jinan, Shandong Province, China

**Keywords:** Severe manifestation, Novel influenza A, Risk factor

## Abstract

**Background:**

No studies on the risk factors of 2009 pandemic influenza A (H1N1) in China have been reported. We aimed to investigate the risk factors for severe manifestations of 2009 pandemic H1N1 influenza in China

**Methods:**

A case–control study with 343 severe hospitalized patients and 343 randomly selected mild controls was conducted. The diagnosis was established by assessment of clinical symptoms and confirmed by the real-time reverse-transcriptase-polymerase chain reaction assay. Severe or mild patients were classified by uniform criteria issued by the Ministry of Health in China.

**Results:**

The multivariable logistic regression analysis showed that the overweight or obese subjects admitted to hospital with H1N1 influenza were more likely to experience severe manifestations. The ORs were 3.70 (95% CI: 2.04-6.72) and 35.61 (95% CI: 7.96-159.21) respectively. Subjects at age less than 5 years or older than 60 years had an increased risk of severe manifestations (OR = 21.14, 95% CI: 7.79-57.33). We also observed increased risk among subjects with longer time interval from symptom onset to hospital admission (OR = 3.26, 95% CI: 2.08-5.11) or peasants (OR = 9.79, 95% CI: 5.11-18.78). Those with chronic disorders had increased risk of severe manifestations of H1N1 influenza.

**Conclusion:**

We provide evidence on the risk factors associated with severe manifestations of 2009 pandemic H1N1 influenza in a study of hospitalized subjects in China.

## Introduction

On June 11, 2009, the World Health Organization raised the pandemic alert level of novel influenza A (H1N1) infection to phase 6, indicating that a global pandemic had begun [1]. The number of severe patients with pandemic H1N1 influenza has increased rapidly. Up to April 25, 2010, the number of confirmed cases in China reached 127,788, with 8347 diagnosed with severe manifestations and 803 deaths, representing a case fatality rate of 0.63% [2]. Although initial reports suggested that the illness that accompanied the 2009 pandemic H1N1 influenza might be milder than the 1918 influenza pandemic, serious complications have been emerging continuously [3,4]. Past studies from western countries have indicated varied risk of severe manifestations of 2009 pandemic H1N1 influenza associated with age, co-existence of chronic diseases, pregnancy, and the time from symptom onset to hospital admission, with particularly elevated risk among elders, infants, pregnant women, subjects with chronic disorders, immunosuppression, or delayed hospital admission [4]–[9]. It holds significance to investigate that whether these factors are associated with 2009 pandemic H1N1 influenza in China. A published study conducted a two-year surveillance of 2009 pandemic H1N1 influenza in Guangzhou, China and focused on the epidemic tendency of H1N1 influenza. This study also evaluated the association between chronic diseases and death of cases with H1N1 influenza [10]. However, no studies on the risk factors of severe manifestations of 2009 pandemic H1N1 influenza in China have been reported. Given the research status, we aimed to investigate whether the major host characteristics and co-existence of chronic diseases are associated with development of severe manifestations of H1N1 infection in China.

In China, all suspicious novel pandemic H1N1 influenza cases were reported to the Chinese Center for Disease Control and Prevention (CDC) through a National Influenza Surveillance Network. The cases were then laboratory-confirmed under the auspices of the Chinese CDC. Among those confirmed patients, cases with severe manifestations were ascertained if satisfying the criteria issued by the Ministry of Health in China (please refer to the Materials and methods section for details). A confirmed case who did not meet any of the criteria was considered to be mild. Information on the major characteristics was collected by using a standardized case report form. We have access to all the hospitalized cases of H1N1 influenza in Shandong Province, including 343 cases ascertained with severe disease and 3639 with mild manifestations.

In this study we investigated the risk factors of severe manifestations of H1N1 influenza in a 1:1 matched case–control study with all these 343 severe patients and 343 randomly selected mild controls.

## Results

Information on the characteristics of study participants is listed in Table 1. We compared the distribution of these variables between cases (severe patients) and controls (mild patients). Cases were more likely to be infants or elders than controls. There was a trend towards a higher prevalence of severe manifestations of pandemic H1N1 influenza in females. Severe cases tended to occur among peasants. We also observed a higher percentage of participants with obesity, pregnancy, and chronic disorders among severe cases. Moreover, severe patients tended to have a longer time before their hospital admission. The variables of age, occupation, body mass index (BMI), time from symptom onset to hospitalization, co-existence of chronic respiratory disease or diabetes, gender and pregnant status reached statistical significance, and were included in the multivariable logistic regression model.

**Table 1:** Characteristics of study participants by severity of manifestations from 2009 H1N1 Influenza, as reported to the Shandong Center for disease control and prevention, China, during the period from May 13, 2009 to March 31, 2010

**Characteristics**	**Severe cases**	**Mild cases**	P^*^
	**n=343**	**n=343**	
**Age (years) (%)**			
≤5	28 (8.2)	5 (1.5)	<0.001
>5 and <60	290 (84.5)	338 (98.5)	
≥60	25 (7.3)	0	
**Gender (%)**			
Female	190 (55.4)	142 (41.4)	<0.001
Male	153 (44.6)	201 (58.6)	
**Ethnic groups (%)**			
Han Chinese	337 (98.2)	339 (98.8)	0.52
Others	6 (1.8)	4 (1.2)	
**Occupation (%)**			
Peasant	138 (40.2)	14 (4.1)	<0.001
Others	205 (59.8)	329 (95.9)	
**Body mass index (kg/m**^**2**^**) (%)**			
<18.5	45 (13.1)	85 (24.8)	<0.001
18.5~24.9	151 (44.0)	233 (67.9)	
25.0~29.9	97 (28.3)	23 (6.7)	
≥30	50 (14.6)	2 (0.6)	
**Chronic coexisting illness (yes, %)**			
Chronic respiratory disease	30 (8.9)	1 (0.3)	<0.001
Diabetes	20 (6.0)	1 (0.3)	<0.001
Chronic renal disease	10 (2.9)	0	0.002
Chronic liver disease	8 (2.4)	0	0.007
Cancer or tumor	6 (1.8)	0	0.03
Chronic cardiac disease	46 (13.5)	0	<0.001
Immunosuppressive disorder	9 (2.9)	0	0.004
**Pregnant status (yes, %)**	72 (21.0)	4 (1.2)	<0.001
**Time from symptom onset to hospital admission (days) (%)**			
≤2	144 (42.0)	229 (66.8)	<0.001
>2	180 (52.5)	83 (24.2)	
Missing	19 (5.5)	31 (9.0)	
**Receiving seasonal influenza vaccination one year before onset (yes, %)**	3 (1.2)	5 (1.7)	0.48

^*^χ^2^ test or Fisher’s exact test.

Multivariable analysis showed that the overweight or obese subjects were more likely to experience severe manifestations of pandemic H1N1 influenza; the ORs were 3.70 (95% CI: 2.04-6.72) and 35.61 (95% CI: 7.96-159.21) respectively, compared with BMI less than 25.0. We observed that subjects aged less than 5 years or older than 60 years had a markedly increased risk of severe manifestations (OR 21.14, 95% CI 7.79-57.33). For age ≤5, the OR (95% CI) was 17.41 (6.30-48.16). For age ≤60, all the subjects were in the group of severe cases. We also found significantly increased risk of severe disease among subjects with a longer time interval from symptom onset to hospital admission (OR = 3.26, 95% CI: 2.08-5.11) or peasants (OR = 9.79, 95% CI: 5.11-18.78). Those with chronic respiratory disease, diabetes and pregnant participants were associated with an increased risk of severe manifestations of H1N1 influenza; the ORs were 42.98 (95% CI 5.24-352.55), 25.51 (95% CI 3.06-212.75), and 8.43 (95% CI 2.68-26.58) respectively (Table 2). We also tested the potential interactions between risk factors on risk of severe manifestations. However, no significant two-way interaction terms were observed.

**Table 2:** Association of selected characteristics with severe manifestations of H1N1 influenza

**Risk factors**	**Univariate analysis**		Multivariable analysis^*^	
	**OR**	**95% CI**	**OR**	**95% CI**
**Age (years)**				
>5 and <60	1.00		1.00	
≤5 or ≥60	12.35	4.87-31.32	21.14	7.79-57.33
**Occupation**				
Others	1.00		1.00	
peasant	15.82	8.89-28.16	9.79	5.11-18.78
**Body mass index (kg/m**^**2**^**)**				
< 25.0	1.00		1.00	
25.0~29.9	6.84	4.20-11.15	3.70	2.04-6.72
≥30.0	40.56	9.76-168.57	35.61	7.96-159.21
**Chronic respiratory disease**				
No	1.00		1.00	
Yes	32.78	4.44-241.79	42.98	5.24-352.55
**Diabetes**				
No	1.00		1.00	
Yes	21.18	2.83-158.70	25.51	3.06-212.75
**Gender/pregnancy**				
Male	0.89	0.64-1.23	0.66	0.43-1.02
Female, no	1.00		1.00	
Female, yes	21.05	7.47-59.34	8.43	2.68-26.58
**Time from symptom onset to hospital admission (days)**				
≤2	1.00		1.00	
>2	3.45	2.47-4.81	3.26	2.08-5.11

^*^ Logistic regression model, adjusting for other risk factors listed in the table simultaneously.

In the secondary analysis, we fitted models by stepwise selection of the variables and the results revealed the best performance of the same model as we used in the primary analysis. A sensitivity analysis was also conducted by enrolling all the variables in the univariate analysis into the model; however, we did not see any material change of the effect estimates for the major variables that were included in the primary model.

## Discussion

In this case control study of 2009 pandemic H1N1 infection among residents in Shandong Province, the pregnant women, those with co-morbidities of chronic diseases, and a longer interval from symptom onset to first visit of hospital had markedly increased risk of severe manifestations of pandemic H1N1 influenza. In addition, peasants had particularly higher risk of severe manifestations compared with other occupational groups, which may hold public health significance.

Li T. et al. reported that the scale and duration of outbreaks, and fatality rates of 2009 pandemic H1N1 influenza were significantly decreased in the second epidemic year compared with those in the first epidemic year (May 2009-April 2010), but similar to those of seasonal influenza [10]. In consistent with the epidemic tendency found in this study, we also chose April of 2010 as the end time of inclusion in our analysis.

Obesity has been previously reported to be associated with severe manifestations of H1N1 influenza [11]–[13]. Our study added to the previous report by comparing severe hospitalized patients with mild patients in China. Even if obesity may be only a proxy measure for other underlying conditions, BMI is an easily obtainable measurement that may be useful for quickly identifying severe patients to target for treatment and prevention measures [4].

In our study, severe manifestations of pandemic H1N1 influenza were associated with the presence of clinical co-morbidities, such as chronic respiratory disease and diabetes. Our results concurred with data from the United States [11,14], which indicated a proportion of more than 70% of H1N1 patients with at least one concomitant chronic disease, such as asthma, other lung diseases, and diabetes, as well the data from a Chinese study, which showed the fatal cases of pandemic H1N1 influenza associated with chronic pulmonary disease [10]. For hospitalized patients of suspected or confirmed influenza with other concomitant complications, current interim guidelines of Chinese CDC for pandemic and seasonal influenza recommend the use of either oseltamivir or zanamivir for treatment [15].

Pregnant women have been reported to exhibit increased risk of seasonal or pandemic influenza and influenza-associated complications in previous reports [16]–[20]. During the 1918 influenza pandemic, the overall disease-specific mortality rate in pregnant women was 27%, suggesting the significance of pregnancy for severe manifestations during the influenza pandemics [16]. One recent report also highlighted the association between pregnancy and the current pandemic of H1N1 influenza, and showed that patients or clinicians may want to avoid antiviral treatment due to concerns about the fetus [6]. Consistent with these studies, pregnancy was associated with severe manifestations of H1N1 influenza in our study. Given the research evidence, clinicians may consider screening pregnant patients with suspected influenza or pneumonia, with the aim of initiating treatment immediately after onset of symptoms. Pregnant and postpartum women should be encouraged to receive vaccination for influenza [21,22].

Past evidences showed that seasonal influenza hospitalizations are more common among persons at advanced ages or under 5 years [23]. In Mexico, a retrospective study found that the disease tended to be more severe in infants and those older than 60 years than in other age-groups [5]. Past evidence also showed that hospitalized patients with pandemic H1N1 influenza aged 50 years or older were most likely to die [4]. Consistent with these evidence, our study found increased risk of severe manifestations of H1N1 influenza among those at advanced age or age under 5 years, suggesting a possible U-shape relationship with age. We observed significantly increased risk for age less than 5 years. For age more than 60 years, all the subjects went to the group with severe manifestations, which supported the higher risk of severe manifestations among those at advanced aged. Clinicians should closely monitor and promptly treat younger or older hospitalized patients with H1N1 infection.

The significant associations of severe manifestations of pandemic H1N1 influenza with pregnancy and other chronic complications are worth further investigation. Pregnant women may be more likely to be admitted for observation even with less severe symptoms than non-pregnant women. Subjects with diabetes or other respiratory diseases might also be more likely to be admitted even with mild influenza-related manifestations; therefore a lower OR for severe manifestations of H1N1 influenza among these subjects could be expected from this perspective. However, previous reports have shown that the pregnant cases tended to deteriorate [6]. It is worth noting that the cases were comprehensively defined as having severe manifestations based on the symptoms presented during the whole disease course, rather than just at time of the admission. Interpretation of the results requires caution.

Peasants were at significantly elevated risk of severe influenza, which may be due to the relatively lower socioeconomic status, limited health awareness and accessibility of rural residents to health care facilities. These data may have implications for public health at the community level. Health education and promotion should be strengthened in rural areas.

This study is limited by its retrospective case–control design of cases only. Cases were drawn from hospitalized inpatients, whereas controls were drawn from patients with mild manifestations of H1N1 influenza. Potential selection and information bias is likely due to the study design. The control selection of mild patients rather than healthy population would have resulted in conservative estimates of ORs for the potential risk factors. Our sample size is only moderate, which limits the further exploration of the associated factors. We lack sufficient power to provide the OR specifically for those at age ≥60 years. However, we included all the cases with severe manifestations of H1N1 influenza in Shandong Province, and selected controls based on a pre-designed 1:1 case control study. The selected controls (mild cases) were very similar in the distribution of the major characteristics with the total 3639 mild cases in Shandong. We cannot evaluate several chronic diseases further except for chronic respiratory diseases and diabetes. However, even among the total 3639 mild patients, we did not find one patient with actively other chronic disorders. Cases with obvious aggravation of previous chronic diseases were assessed as severe cases. The frontline health professionals therefore could tend to diagnose those with concomitant chronic disorders as severe cases. As an observational study, we cannot rule out the possibility of residual confounding by unmeasured or imperfectly measured risk factors. We lacked information on some potentially important factors, such as the history of close contact and medication uses.

## Conclusions

In conclusion, our case control study has provided evidence on the risk factors associated with severe manifestations of hospitalized patients with pandemic H1N1 influenza in China. Further studies are warranted to confirm our findings.

## Materials and methods

### Data sources

In China, all suspicious 2009 pandemic H1N1 influenza cases are reported to the Chinese CDC through a National Influenza Surveillance Network. Throat swab specimens were collected for each participant, and then sent to the Network. Cases were then laboratory-confirmed by the positive results on a real-time reverse-transcriptase-polymerase chain reaction assay performed under the auspices of the Chinese CDC.

Among these confirmed patients, cases with severe manifestations were defined as having at least one of the following symptoms: 1) persistent fever for >3 days; 2) severe coughing with purulent sputum, bloody sputum, or chest pain; 3) rapid breathing, breathing difficulty and lip cyanosis; 4) altered mental status, such as slow response, hypersomnia, dysphoria and convulsions; 5) heavy vomiting, diarrhea and signs of dehydration; 6) signs of pneumonia in imaging examinations; 7) rapidly elevated levels of cardiac enzymes such as creatine kinase and creatine kinase-MB increasing rapidly; and 8) the obvious aggravation of previous chronic diseases [15]. A confirmed case who did not meet any of the above criteria was considered to be a mild patient. We collected information on the main demographic variables (age, sex, occupation and ethnic groups), BMI (defined as weight divided by square of height, kg/m^2^), time from symptom onset to hospital admission, receiving seasonal influenza vaccination one year before onset of H1N1 influenza (yes or no), co-existence of chronic diseases (chronic pulmonary diseases, diabetes, renal diseases, chronic cardiac disease, immunosuppressive disorder, or cancer) and pregnant status (yes or no) in occurrence of H1N1 influenza. Chronic pulmonary diseases included asthma, chronic bronchitis, tuberculosis, emphysema, chronic obstructive pulmonary disease and tuberculosis pleurisy. All these information was collected by a standardized case report form issued by the Ministry of Health in China. This form was applied to each case within 24 h after the clinical outcome (disease cure or death). All case report forms were filled by the frontline physicians in the hospitals responsible for diagnosis and treatment of H1N1 influenza designated by the local CDC in Shandong Province. The face-to-face interviews were conducted, and information from the medical records was reviewed to help double-check the surveys. All the surveyors were strictly trained for the survey, to ensure that they conducted the interviews according to uniform standards and methods.

We have access to all the patients of H1N1 influenza in Shandong Province. Totally we identified 3982 cases between May 13, 2009 and March 31, 2010, by China novel influenza A (H1N1) surveillance system from Shandong Province. We chose April of 2010 as the end time of inclusion because the incidence of H1N1 influenza in China began to fall to a low level since then with less than 30 confirmed cases per week. A total of 343 cases were ascertained with severe disease, while 3639 with mild manifestations. Random number method was applied to sample 343 from the total cases with mild manifestations. These were included as controls in our study. We have the complete data for most of the major characteristics. For time from symptom onset to hospital admission, we have information for 324 cases of severe manifestations (94.5%), and 312 of mild manifestations (91.0%). We found very similar distribution of the major demographic factors between all the 3639 mild patients and these selected 343 controls.

### Statistical analysis

According to the distribution of the characteristics, the occupation status was reclassified into two categories (peasant or others). BMI was classified into four categories [underweight (<18.5 kg/m^2^), normal (18.5~24.9 kg/m^2^), overweight (25.0~29.9 kg/m^2^), or obese (≥30 kg/m^2^). Time from symptom onset to hospitalization was classified as two categories (≤2 or >2 days). For initial screening of potential risk factors, we applied univariate analysis by using *χ*^2^ test or Fisher’s exact test to compare the distribution of different variables in the above-mentioned categories between severe and mild patients, including age, sex, ethnic groups, occupation, BMI, time from symptom onset to hospitalization, chronic coexisting illness, pregnancy status, and seasonal influenza vaccination one year before onset.

We compared the distribution of each characteristic between cases and controls. For variables reaching statistical significance (*P* <0.05), we then carried out univariate and multivariable-adjusted analysis to estimate the odds ratio (ORs) and corresponding 95% confidence intervals (CIs) for associations of potential risk factors with severe manifestations of H1N1 influenza. Among patients with renal disease, chronic liver disease, chronic cardiac disease, cancer or immunosuppressive disorders, we found no participants with mild H1N1 influenza. Therefore we did not include these chronic diseases in the multivariable regression analysis. For participants aged more than 60 years, there are no subjects in the control group. Therefore for the variable age, we combined the category “<5 years” with “≥60 years”, given the past evidence which showed a higher risk of severe influenza in infants and elder people. To evaluate the risk for overweight or obese subjects, the BMI category of “underweight” was combined with “normal”. We derived a new variable to merge sex and pregnancy status to avoid co-linearity of the model, with three categories (men, women without pregnancy, or women with pregnancy) and women without pregnancy as the reference group. For the variables with missing data (time from symptom onset to hospital admission), we created an indicator variable for the missing category.

To check the validity of the model, we fitted models by forward stepwise selection of the variables. We also conducted a sensitivity analysis by enrolling all the variables in the univariate analysis into the multivariable regression model.

We conducted all statistical analyses using SAS software (v9.1; SAS Institute Inc, Cary, NC). All statistical tests were 2-tailed, and the significance level was set at *P* <0.05.

The study was approved by the Institutional Review Board at the Shandong University School of Public Health. All participants had been aware that information was being collected for the purpose of future series of research and provided written informed consent for participating in these studies. Therefore for the current specific analysis, the Institutional Review Board waived the need for further consent to participate.

## Abbreviations

BMI: Body mass index; CI: Confidence interval; CDC: Center for Disease Control and Prevention; OR: Odds ratio.

## Competing interests

The authors declare that they have no competing interests.

## Authors’ contributions

XJW and ZQB have full access to all of the data in the study and take responsibility for the integrity of the data and the accuracy of the data analysis. XJW and ZQB conceived of the study. YYR, YYY, WQL, XJW and ZQB participated in its design and coordination. XJW and ZQB acquired the data. YYY performed the statistical analysis. YYR, YYY, WQL, YL, TL, SW, SZ, and ZL prepared the first draft of the manuscript while YYR and WQL made critical revisions to it. All authors read and approved the final manuscript.
